# Changes in chest wall motion with removal of Nuss bar in repaired pectus excavatum – a cohort study

**DOI:** 10.1186/s13019-018-0827-1

**Published:** 2019-01-08

**Authors:** Nicola Oswald, Zara Jalal, Salma Kadiri, Babu Naidu

**Affiliations:** 1 Institute of Inflammation and Ageing, College of Medical and Dental Sciences, Centre for Translational Inflammation Research, University of Birmingham Laboratories, Queen Elizabeth Hospital Birmingham, Edgbaston, Birmingham, B15 2TT UK; 2 Department of Thoracic Research, Heartlands Hospital, Birmingham, B9 5SS UK

**Keywords:** Pectus excavatum, Nuss procedure, Chest wall motion, Optoelectronic plethysmography

## Abstract

**Background:**

The effects of the Nuss procedure on chest wall motion and spirometry have previously been described; we aimed to describe the effects of removal of the Nuss bar.

**Methods:**

We studied 9 patients just prior to and 6 weeks after Nuss bar removal. Regional chest volume changes, synchrony of respiratory movement and spirometry were recorded using optoelectronic plethysmography (OEP) and compared. Recordings were performed at rest and exercise during cycle ergometry.

**Results:**

There were small but statistically significant changes in tidal volumes of the diaphragmatic ribcage compartment during exercise (+ 48 ml, *p* = 0.038, Cohen’s d = 0.12) and percentage contribution of the diaphragmatic ribcage to total tidal volumes at rest (+ 2.7 percentage points, p = 0.038, Cohen’s d = 0.12). Synchrony of respiratory movements at rest and during exercise was unchanged following Nuss bar removal. There were no significant changes in spirometry and exercise capacity.

**Conclusions:**

The effects of Nuss bar removal on diaphragmatic ribcage motion are detectable but small and unlikely to be of clinical significance. No change in exercise capacity should be expected after Nuss bar removal.

**Trial registration:**

Registered at ClinicalTrials.gov, identifier NCT02958683, registered 5th August 2016, first patient enrolled July 2016, retrospectively registered.

## Background

Pectus excavatum (PE) is the most common chest wall deformity with a prevalence of approximately 12 per 10,000 persons [1]. The most commonly used surgical treatment at our institution is now the Nuss procedure; this has provided high patient satisfaction which is also apparent anecdotally to the clinicians managing this patient group [2, 3]. Evidence is mounting that repair of severe PE improves cardiopulmonary function however controversy persists over the physiological effects of surgical correction, with opponents considering this a purely cosmetic procedure. Having previously described the effects of Nuss bar insertion on patients’ chest wall motion up to 6 months postoperatively, in this study we aimed to describe the effects of Nuss bar removal after correction and so between the two studies provide a more complete picture of changes in chest wall function induced by the procedure [4]. We hypothesised that chest wall motion would improve after Nuss bar removal compared to with the bar in situ; this would be demonstrated by a lower respiratory rate to achieve the same minute volume with the increase in tidal volume attributable to the diaphragmatic ribcage region.

## Methods

### Participants

Nine male patients aged 17 to 23 years were recruited 2 years following the Nuss procedure to treat PE in a prospective, single centre cohort study. All patients listed for removal of one or more Nuss bars during the study period (12 months) were eligible; patients were excluded if they were having further surgical procedures to manage their pectus excavatum at the time of bar removal (one patient excluded). Patients underwent removal of their Nuss bar as a day case procedure under general anaesthetic and were followed up clinically at 6 weeks. The recruitment rate was 81.8%, all patients who underwent preoperative testing completed follow up and there was no missing data.

### Study investigations

**Fig. 1:**
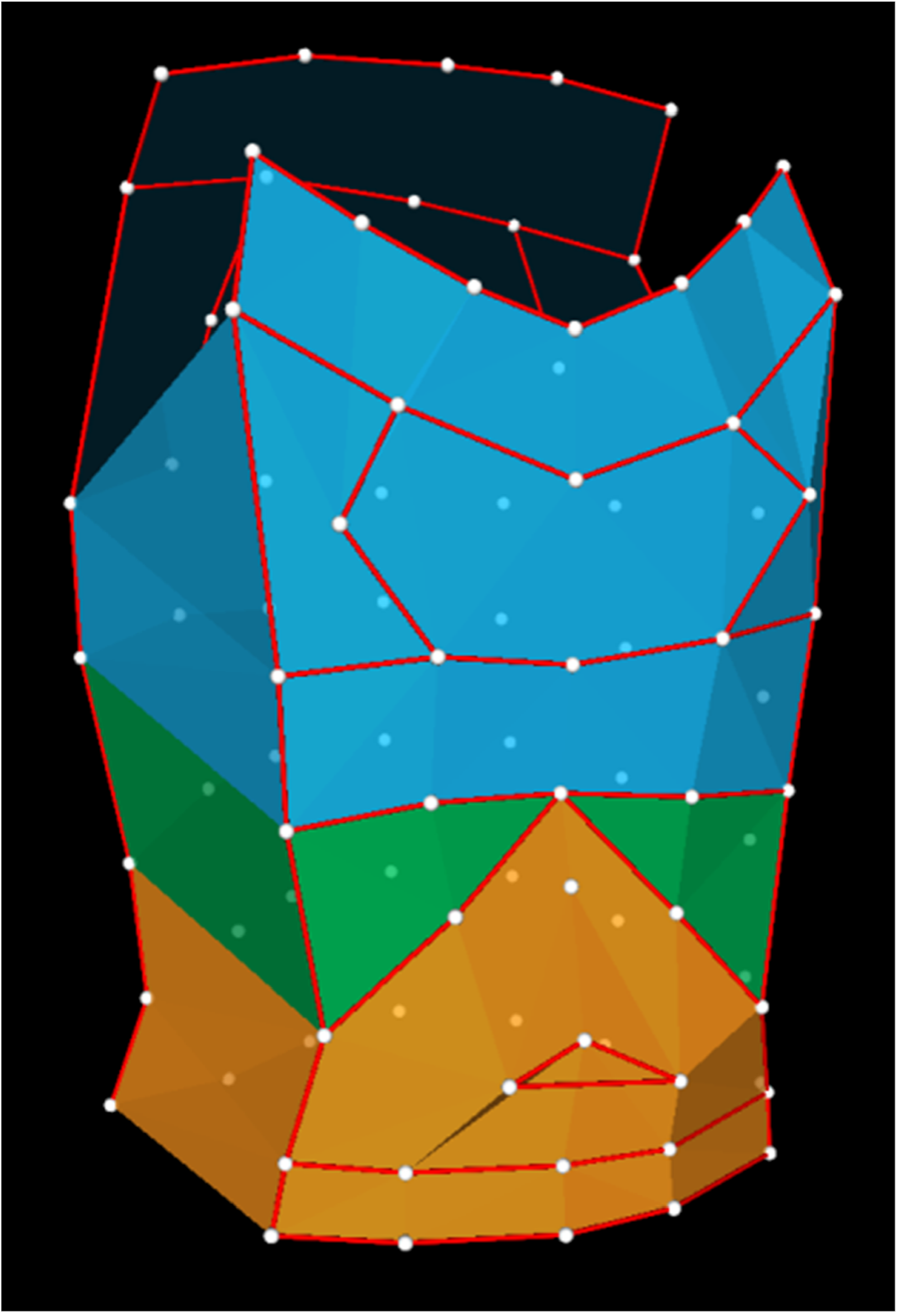
Image of marker reconstruction using OEP showing divisions of upper ribcage (blue), diaphragmatic ribcage (green) and abdomen (orange)

Chest wall motion was recorded using optoelectronic plethysmography (OEP; BTS, Milan, Italy) once at the time of preoperative assessment and again at 6 weeks following bar removal. Details of the technique have been reported previously [5]. In summary, 89 reflective markers are placed on the patient’s torso according to anatomical landmarks and infrared light is both projected and recorded by 8 surrounding cameras. OEP was performed by the same researcher for all patients at each visit to minimise bias due to interobserver variation [6]. The positions of the markers on the chest are recorded and reconstructed into a three-dimensional model using SMART suite software (BTS). The model assesses the trunk in three compartments: the upper ribcage that is apposed to the lung, the lower ribcage that is apposed to the diaphragm (hereafter referred to as diaphragmatic ribcage), and the abdomen (Fig. 1). The tidal volumes and synchrony of movements between each compartment can then be derived. OEP was performed during 2 min of quiet breathing with a vital capacity manoeuvre at 60 s followed by incremental cycle ergometry up to 80% of the patient’s predicted maximum heart rate (220 – age × 0.8 beats per minute). Spirometry was performed prior to chest wall motion analysis (Vitalograph 2150, Ennis, Ireland).

### Analysis

Statistical analysis was performed using SPSS version 24 (IBM Corp, New York, USA). Shapiro Wilk testing and histogram inspection were applied to assess data for normality; paired T tests or Wilcoxon Sign rank tests were applied if data were normal or not normal in distribution respectively, *p* < 0.05 was considered statistically significant. Effect sizes for non parametric tests were assessed as small (0.1), medium (0.3) and large (0.5); effect sizes for parametric tests were assessed as small (0.2), medium (0.5) and large (0.8) according to Cohen’s criteria [7]. Comparison of dichotomous data was performed using Fisher’s exact test. A sample size of 9 was required to detect a 50 ml difference in tidal volumes at the diaphragmatic ribcage with a power of 80% for a 2 sided test and a type I error rate of 5% (assuming mean 275 ml preoperatively, mean 350 ml postoperatively and 50 ml standard deviation). The mean difference of repeated measures of chest wall volume using OEP is 30 ml (+/− 11 ml) for absolute volumes and 1.5 percentage points (+/− 5.1) for percentage contributions [6].

## Results

The baseline characteristics of patients are shown in Table 1; spirometry data before and after bar removal are shown in Table 2.

**Table 1:** Baseline patient characteristics, *n* = 9

Age at bar insertion, years mean (SD)	17 (2)
Age at bar removal, years mean (SD)	19 (2)
Height, metres mean (SD)	1.8 (0.09)
Weight, kilograms mean (SD)	65.8 (12.1)
Body Mass Index, mean (SD)	20.0 (2.8)
Pectus shape, n (%)	
Cup	5 (55.6)
Saucer	3 (33.3)
Mixed	1 (11.1)
Haller index before correction, mean (SD)	5.1 (1.1)

**Table 2:** Spirometry and exercise time

	Bar in situ Mean (SD)	Bar removed Mean (SD)	Mean difference (95% Confidence Interval)	Effect size	p
FEV_1_ (L)	4.12 (0.67)	4.24 (0.78)	0.12 (−0.23–0.46)	0.261	0.456
FEV_1_% predicted	87.9% (16.9)	90.6% (19.7)	2.70 (−5.14–10.54)	0.265	0.450
FVC (L)	4.59 (0.90)	4.68 (0.81)	0.09 (−0.22–0.40)	0.220	0.528
FVC % predicted	82.0% (14.8)	84.8% (15.3)	2.78 (−0.30–8.55)	0.370	0.299
FEV_1_/FVC	90.7% (6.8)	90.9% (8.0)	0.24 (−5.22–5.71)	0.034	0.921
Time to 80% max heart rate	451 (128)	425 (137)	−27 (− 143–90)	0.211	0.597

In our group there was no significant difference in spirometry in absolute volumes or in percentage predicted volumes. There was also no effect on Forced Expiratory Volume in 1 s to Forced Vital Capacity (FEV_1_/FVC) ratio or time to reach 80% maximum heart rate.

### Chest wall motion at rest

Tidal volumes and respiratory rate were unchanged by bar removal. There was a statistically significant increase in percentage contribution of the diaphragmatic ribcage following bar removal but the effect size was small (Table 3).

**Table 3:** Chest wall motion at rest (median and IQR)

	Bar in situ	Bar removed	Effect size	*p* value
Respiratory rate	15 (13–17)	16 (12–17)	0.003	0.953
Tidal volume (Vt, ml)	668 (503–784)	682 (538–920)	0.049	0.374
Vt ribcage	315 (187–384)	307 (214–361)	0.003	0.953
Vt diaphragm	73 (66–119)	96 (75–154)	0.095	0.086
Vt Abdomen	257 (220–324)	257 (224–301)	0.003	0.953
% ribcage	42.7 (34.5–52.0)	41.6 (34.1–51.1)	0.030	0.594
% diaphragm	12.9 (11.7–15.8)	15.6 (14.2–18.9)	0.115	0.038
% abdomen	38.9 (34.4–52.0)	44.7 (33.9–50.9)	0.003	0.953

The change in absolute volumes contributed by the diaphragmatic ribcage approached statistical significance and the effect size approached a small increase. There was no significant difference in synchrony of chest wall motion at rest (*p* = 0.471).

### Chest wall motion during exercise

**Fig. 2:**
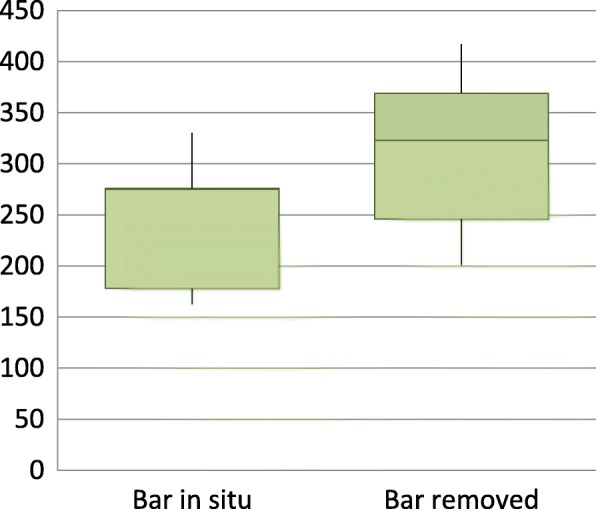
Tidal volume of diaphragmatic ribcage during exercise during exercise (ml)

**Fig. 3:**
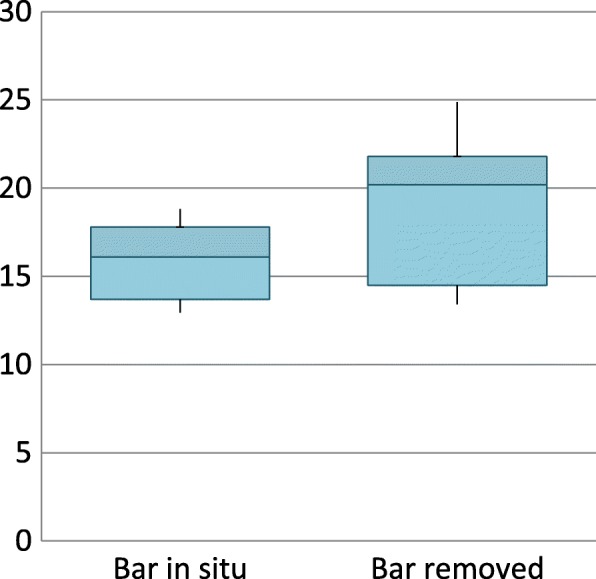
Contribution of diaphragmatic ribcage to tidal volumes during exercise (%)

Tidal volumes and respiratory rate were also unchanged by bar removal during exercise. There was a statistically significant increase in the absolute tidal volumes of the diaphragmatic ribcage but again the effect size was small (Table 4 and Fig. 2). The change in percentage contribution of the diaphragmatic ribcage approached statistical significance and had a small effect size (Fig. 3). There was no significant difference in synchrony of chest wall motion during exercise (*p* = 0.999).

**Table 4:** Chest wall motion during exercise at 80% maximum heart rate (median and IQR)

	Bar in situ	Bar removed	Effect size	p value
Respiratory rate	32 (28–37)	34 (24–36)	0.089	0.110
Tidal volume (Vt, ml)	1555 (1288–1731)	1643 (1384–1847)	0.089	0.110
Vt ribcage	628 (455–655)	629 (577–760)	0.076	0.173
Vt diaphragm	275 (178–275)	323 (246–369)	0.115	0.038
Vt Abdomen	771 (624–864)	708 (473–708)	0.049	0.374
% ribcage	37.2 (31.3–40.2)	37.4 (34.4–44.3)	0.049	0.374
% diaphragm	16.1 (13.7–17.8)	20.2 (14.5–21.8)	0.109	0.051
% abdomen	49.1 (45.0–53.5)	42.3 (33.8–50.4)	0.076	0.173

## Discussion

This study contributes towards understanding of the impact of Nuss repair of pectus excavatum upon respiratory function by describing the later changes in chest wall motion, specifically whether the Nuss bar restricts chest wall motion as has been reported in the early phase after insertion [4]. The results indicate there is a detectable restriction of function at the level of the diaphragmatic ribcage both at rest and during exercise that is reversed by bar removal, but this restriction is small. A restriction in motion at the diaphragmatic ribcage is intuitive because bar positioning is most commonly at the caudal portion of the sternum so any changes might be expected to affect this part of the trunk. Overall this means that the rigid bar inserted during the Nuss procedure has little impact upon chest wall motion and respiratory function at 2 years after insertion.

Improvement in exercise capacity is reported after insertion of a Nuss bar to repair PE; this has been shown to be related to improved ventricular filling as the heart is relieved of compression by the displaced sternum [8–10]. The impact of PE upon respiratory mechanics is more controversial with inconsistent results of both static lung function and non invasive assessment of chest wall motion [4, 11]. Chest wall motion analysis using OEP at our centre has shown that the increase in exercise tolerance following insertion of a Nuss bar is not due to increased tidal volumes, in fact there was restriction in tidal volumes in the early postoperative period that approached but did not reach baseline at 6 months [4]. Compartmental tidal volumes were found to be unaltered by the Nuss procedure during maximum voluntary ventilation at another centre [12]. Analyses that focussed on movements of the visibly depressed portion of the chest wall (the anterior midline) during maximum voluntary ventilation did however show a significant reduction in movement in PE compared to controls and significant changes following the Nuss procedure; movement of the depressed sternum increased and abdominal excursion decreased after the Nuss procedure to match the movement pattern of normal controls [11, 13].

The available literature together suggests that the distribution of chest wall motion during extreme respiratory manoeuvres is altered by the Nuss procedure but this does not underlie changes in exercise tolerance or result in increased tidal volumes. Interpreting our results in this context may imply that the restriction in chest wall motion early after correction may be due to soft tissue tension; this soft tissue tension then lessens over time as the thoracic structures adjust to their new position leaving only a minimal restriction due to the Nuss bar alone.

The key weakness of this study is that we were unable to study to same patients both prior to any surgical correction of PE and after removal of the Nuss bar, so we do not know if there was any change in the reported measures between these two points for individual patients. However, comparison of exercise tolerance over 3 years would be subject to confounding due to changes in lifestyle and growth. It is also possible that the aerobic capacity of participants may have changed over the 6 weeks of follow up by a change in exercise regime. None of the participants reported chest pain at the first visit or follow up visit. Another weakness is the relatively small number of patients studied; although we met the required sample size the results may not be generalisable for example to female patients or paediatric patients. Patients undergoing the Nuss procedure at our centre are typically in their late teenage years; other centres or other countries may routinely operate on younger patients. It is not known whether pre-adolescent patients have different changes in chest wall motion when treated with the Nuss procedure.

## Conclusions

Our results are consistent with available data in that changes of chest wall function in PE are present but small; the predominant benefit of correction of PE still appears to be due to better cardiac function. Clinically this means that further improvement in exercise capacity should not be expected following removal of Nuss bars. The presence of a rigid bar traversing the anterior chest wall only has a small effect on chest wall motion in the late postoperative period.

## Abbreviations

FEV1: forced expiratory volume in 1 s
FVC: forced vital capacity
OEP: optoelectronic plethysmography
PE: pectus excavatum
REC: research ethics committee
